# Enhancement of Hip X-ray with Convolutional Autoencoder for Increasing Prediction Accuracy of Bone Mineral Density

**DOI:** 10.3390/bioengineering10101169

**Published:** 2023-10-08

**Authors:** Thong Phi Nguyen, Dong-Sik Chae, Sung Hoon Choi, Kyucheol Jeong, Jonghun Yoon

**Affiliations:** 1 Department of Mechanical Design Engineering, Hanyang University, Seoul 04763, Republic of Korea; npthong2511@hanyang.ac.kr (T.P.N.); aquapura@hanyang.ac.kr (K.J.); 2 BK21 FOUR ERICA-ACE Center, Hanyang University, Ansan 15588, Republic of Korea; 3 Department of Orthopedic Surgery, International St. Mary’s Hospital, Catholic Kwandong University College of Medicine, Incheon 22711, Republic of Korea; drchae@ish.ac.kr; 4 Department of Orthopaedic Surgery, Hanyang University College of Medicine, Seoul 04763, Republic of Korea; spineshchoi@hanyang.ac.kr; 5 Department of Mechanical Engineering, Hanyang University, Ansan 15588, Republic of Korea; 6 AIDICOME Inc., Ansan 15588, Republic of Korea

**Keywords:** bone mineral density, radiographs, osteoporosis, autoencoder

## Abstract

It is very important to keep track of decreases in the bone mineral density (BMD) of elderly people since it can be correlated with the risk of incidence of major osteoporotic fractures leading to fatal injuries. Even though dual-energy X-ray absorptiometry (DXA) is the one of the most precise measuring techniques used to quantify BMD, most patients have restricted access to this machine due to high cost of DXA equipment, which is also rarely distributed to local clinics. Meanwhile, the conventional X-rays, which are commonly used for visualizing conditions and injuries due to their low cost, combine the absorption of both soft and bone tissues, consequently limiting its ability to measure BMD. Therefore, we have proposed a specialized automated smart system to quantitatively predict BMD based on a conventional X-ray image only by reducing the soft tissue effect supported by the implementation of a convolutional autoencoder, which is trained using proposed synthesized data to generate grayscale values of bone tissue alone. From the enhanced image, multiple features are calculated from the hip X-ray to predict the BMD values. The performance of the proposed method has been validated through comparison with the DXA value, which shows high consistency with correlation coefficient of 0.81 and mean absolute error of 0.069 g/cm^2^.

## 1. Introduction

Osteoporosis, a prevalent metabolic bone disorder, is distinguished by the gradual depletion of bone mass density. In the context of healthy bones, their structure exhibits a pattern reminiscent of a honeycomb, featuring voids and spaces in proportion to the bone tissue, a vital configuration for maintaining proper bone density. On the occurrence of osteoporosis, holes and spaces in the bone structure increase, which can be attributed to the faster rate of bone tissue loss than that of bone formation. Consequently, bones become less dense, weaker, and more susceptible to fractures, particularly the hip bone. The recovery process for such injuries is challenging, often resulting in patients being unable to lead independent lives. Based on the research of Lyritis et al. [1], mortality rates increase significantly to 28% during the first year, and approximately 44% of patients experience a decline in functional capacity in the second year. Concerning the patients who survived more than one year, Myers et al. [2] and Poor et al. [3] show that only 41% of injured cases can achieve full recovery, with most of the survivors relying on a cane or walker. Approximately 97% of hip fracture cases have osteopenia, and half of them have osteoporosis [4].

To mitigate the impact of osteoporosis and proactively prevent hip fractures, the estimation of bone mineral density (BMD), a crucial indicator of bone health, assumes a pivotal role in osteoporosis diagnosis. Low BMD exhibits a strong association with osteoporosis. Historically, before the advent of modern radiographic imaging techniques for the human body, the Singh index [5] enjoyed widespread use in osteoporosis diagnosis. This index was proposed based on a study that compared and distinguished hip radiographs of normal individuals and those with osteoporosis. In the study, Singh et al. proposed six categories of trabecular pattern according to radiographs of hips and their relationships with osteoporosis. However, this method highly depends on the decision of the expert, which can easily be biased. Although many studies indicate the low accuracy of the Singh index [6,7,8,9], it is still popularly used for osteoporosis diagnosis, especially in local hospitals and medical centers where modern machines and techniques are infeasible. 

When considering approaches that utilize radiation for the internal structural assessment of bone, the measurement of X-ray absorption within the bone using dual-energy X-ray absorptiometry (DXA) has been endorsed by the World Health Organization [10] as a prominent densitometry technique and remains the most prevalent method. However, it is noteworthy that access to DXA scanners can be limited due to their high cost. A similar constraint pertains to the quantitative computed tomography (QCT) method, which translates the Hounsfield units from CT images into BMD values via calibration standards. Nevertheless, it is essential to acknowledge that the QCT method, while serving as a valuable complement to DXA, allows comprehensive assessments of bone geometry and compartments.

Singh’s research and its application proves the relationship between the trabecular patterns on X-rays of hips and their possibility of osteoporosis. However, with the limitation of technology, the diagnosis can be based on six types of patterns, which heavily depend on the doctor’s decision and can easily be biased. With the revolution in machine vision, including pattern recognition and artificial intelligence, analysing hip X-rays and extracting highly correlated bone density features for diagnosis of osteoporosis and BMD prediction is a potential research direction. Pulkkinen et al. [11] have introduced a compelling regression approach that evaluates bone mineral density (BMD) by considering factors such as trabecular structure, density, and bone geometry. Their methodology relies on projectional radiographs and employs a gradient-based image processing technique. In a different vein, Liu et al. [12] have developed a convolutional neural network (CNN)-based method for bone segmentation and BMD estimation using chest X-ray images, particularly for critical infants. Nguyen et al. [13] have introduced a novel approach for predicting hip BMD from X-rays, leveraging a convolutional neural network (CNN). This study employs the Sobel algorithm to extract binary images of trabecular structures from citations 1, 2, and 3 of Singh’s work, subsequently utilizing these images to train regression CNN models for BMD prediction. In a similar vein, Sato et al. [14] focused on BMD prediction from chest X-rays, with an emphasis on expanding the dataset to enhance the performance of their approach.

This research direction has a great potential to fill the gap in medical clinics that lack access to expensive DXA and QCT equipment, providing the doctors with an alternative option for osteoporosis diagnosis and treatment. However, despite the variety of limitations, the number of research endeavors applying artificial intelligence is not commensurate to its potential.

Therefore, this study aims to provide an automatic solution for evaluating the BMD through a hip X-ray. The main novel point of this research is to utilize a convolutional autoencoder model, which is trained via proposed synthesis data, that enhances the quality of the input X-rays by reducing the effect of soft tissue. The enhanced image is then continuously loaded to a proposed landmark detection model system to segment three regions of hips, which are finally used for extracting the grayscale features for measuring BMD. To validate the performance of the proposed method, the predicted BMD was compared to the ground truth values from the DXA machine. In addition, the efficiency of the developed enhancing method for input image is verified by comparing the results obtained using enhanced images to those generated using the original images. The novelty of this research is characterized by the following key points:

- The development of a cost-effective smart system utilizing X-ray technology as a viable alternative to the expensive DXA equipment;
- The implementation of an innovative enhancement method to mitigate the influence of soft tissues, thereby enhancing the precision of bone mineral density (BMD) measurement;
- The noteworthy achievement of remarkable accuracy, underscored by a robust correlation with DXA measurements;
- The broadening of accessibility to BMD measurements, thereby facilitating the early diagnosis of osteoporosis.

## 2. Materials

This study received ethical approval from the Institutional Review Boards (IRBs) of both the Hanyang University College of Medicine (IRB FILE No: 2023-04-016) and the Catholic Kwandong University College of Medicine (IS22RISI0029). The dataset employed in this research comprised 673 X-rays of the hip, each with a pixel resolution of 900 × 900 pixels, cropped from the coronal views of different patients. These images were collected between January 2021 and July 2022 at the International St. Mary’s Hospital, Catholic Kwandong University College of Medicine, Incheon, Republic of Korea. Additionally, the dataset included 266 X-rays of the hip with the same pixel resolution, collected between September 2022 and January 2023 at the Hanyang University Medical Center, Seoul, Republic of Korea. Only high-quality contrast images with suitable brightness were selected for analysis. During the dataset development process, our focus was on female patients due to a significant discrepancy in the number of cases between males and females. This trend aligns with the statistics presented by Newton-John and Morgan [15], which highlight a notably higher percentage of female patients diagnosed with osteoporosis and hip fractures within the same age group. The ground truth BMD data for the dataset was obtained using a DXA scanner (HOLOGIC, Discovery W model), with four BMD values recorded after one round of DXA examination, including those for the femoral neck, trochanter, Ward’s triangle, and the total region. Experts recommended recording the BMD from the neck region, which typically exhibits faster reduction compared to other regions.

Taking into account the influence of biological parameters on BMD, as elucidated by Ambrus et al. [16] and Keaveny and Yeh [17], factors such as genes, sex, age, height, weight, and physical activity were evaluated. However, it is worth noting that genetic and physical activity research demands advanced techniques and prolonged monitoring periods, making them unsuitable for inclusion in this study. As such, this research focused on gender, age, height, and weight as the biological parameters of BMD assessment. Table 1 provides a summary of patient baseline characteristics categorized by gender.

## 3. Methods

**Figure 1:**
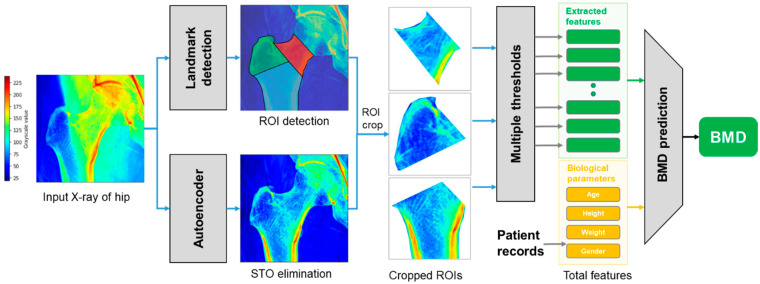
Flowchart of the proposed method for measuring BMD on X-ray of hips.

For achieving the highest prediction accuracy of BMD from hip X-rays, the proposed method first aimed to address the common inevitable challenge associated with using X-ray images, which is the interference of soft tissue in the visualization of bone tissue. This difficulty is overcome using the convolutional autoencoder, which filters the layer of bone tissue from the input X-ray. In addition, three regions of hips are segmented from the input X-ray, which are used for calculating multiple features representing BMD values. These features are finally considered as the input to a machine learning regression model, which outputs BMD values corresponding to the hip, as shown in Figure 1.

### 3.1. Synthesis Data for Enhancement Method

X-ray images, which are considered to be inputs for the developed function, are created by passing one X-ray beam through the human body. Based on the X-ray absorption by different tissues in the patient body, the obtained radiograph enables users to visualize internal body parts by differentiating the dense tissues with the brighter grayscale values from the less dense tissues. Therefore, the main usage of the X-ray image is for monitoring and detecting fractures, tumors, and other abnormalities in bones. 

However, for measuring BMD with the DXA method, the BMD can be measured based on the X-ray absorption of bone tissue, only after separating the absorption of soft and bone tissues using the dual X-ray beam with different energies. In particular, the lower energy beam is more readily absorbed by soft tissue, while the higher energy one aims for the bone tissue. By measuring the difference in attenuation between the high-energy and low-energy X-ray beams, DXA can estimate the BMD of the scanned region.

**Figure 2:**
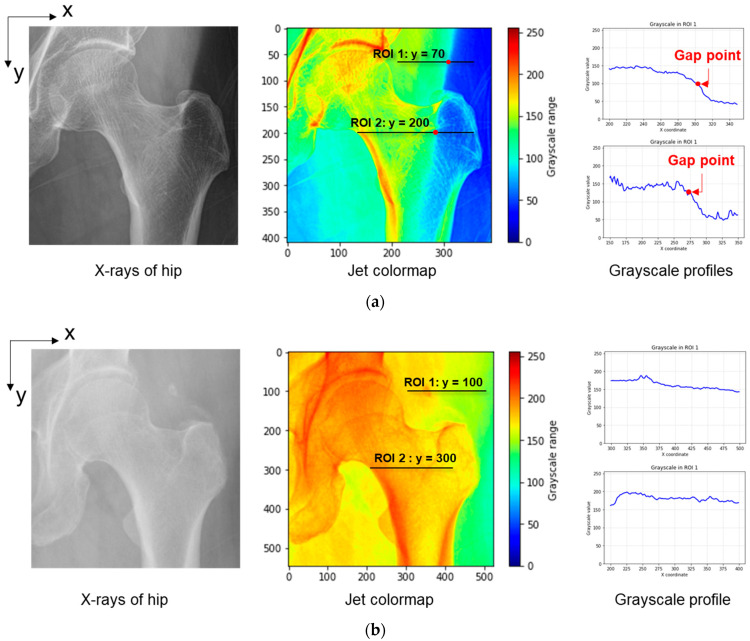
Examples of conventional X-rays of hips: (**a**) Under the uneven distribution of soft tissue amount, which causes gaps (Type 1); (**b**) Under temporarily even distribution of soft tissue amount (Type 2).

The conventional X-rays, on the other hand, only use one single X-ray beam with constant energy during capturing process. Therefore, depending on regions, the grayscale value in the obtained X-rays represents either the absorption of soft tissue or a combination of soft and bone tissues. Also, as shown in Figure 2, the distribution of amount of soft tissue highly affects to the quality of visualization in X-rays of hips. In Figure 2a, there is a gap between the amount of soft tissue in two regions, which caused a boundary. With the division of grayscale values caused by the difference of soft tissue between regions, this type 1 X-ray of hips is considered to be having a high effect on extracting features for measuring BMD. On the other hand, with cases temporarily having even distribution of soft tissue amount, as shown for type 2 in Figure 2b, it is more efficient for the process to analyze features.

And the aim of utilizing a deep learning-based method is to separate the absorption of soft tissues from the input X-ray. Similar to other supervised learning methods, to train the deep learning model, a dataset was required with two parts: the input images, which are the original X-ray images of hips, and the label images, which are the images of soft tissue-only absorption (STO). However, collecting both types of images on one scanned region is not possible using conventional single-beam X-ray device. So, an idea was proposed to generate a synthesis dataset, which has the same features as those required for training tasks. 

**Figure 3:**
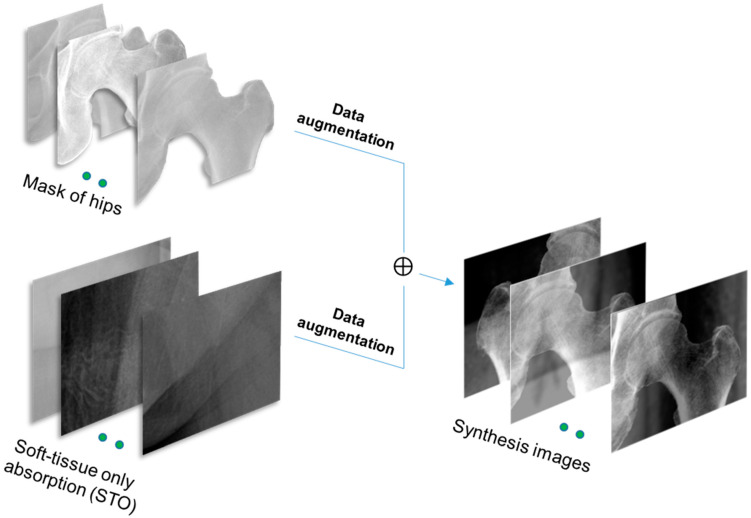
Procedure for generating synthesis images for training autoencoder model.

In particular, the STOs were first collected from multiple regions on the X-ray of the lower limbs, where there was no part of bone involved, as demonstrated by the blue boxes on Figure 3. Here, there were 2800 STO images. Secondly, the ROIs of hips, which are represented by the yellow box in Figure 3, are collected. Considering the collected ROI images of hips, cases that show the significant impact of soft tissue with complex grayscale distribution and interrupted boundaries caused by different volume of soft tissue mass as type 1 were excluded. Only X-ray of hips which were visually classified into type 2 were selected. In total, there were 180 masks of hips collected. Thirdly, from the collected hip images, the area of a hip was segmented from the original image, which was finally projected onto the collected STO to generate the synthesis image, as shown in Figure 3. To increase the diversity of the dataset, an augmentation step was implemented prior to projection, which included the rotation and flipping of the hip mask. 

Finally, the set of obtained synthesis images serve as the input data, similar to a conventional hip X-ray, while the corresponding STOs are utilized as the label images to train a deep learning model.

### 3.2. X-ray Enhancement with Convolutional Autoencoders

The purpose of this enhancement is to reduce the effect of soft tissue on the input X-rays of the hip. This step is taken using the autoencoder technique. Autoencoders are neural networks initially used in unsupervised learning tasks, such as dimensionality reduction, feature extraction, and data compression. The incorporation of Autoencoders offers several distinct advantages. Firstly, Autoencoders enable effective dimensionality reduction, making them particularly valuable for high-dimensional datasets. Secondly, they excel in feature extraction, helping us to identify critical patterns within complex data. Additionally, autoencoders aid in anomaly detection by sensitizing the model to irregularities, which is crucial for our study. Their ability to capture non-linear relationships is advantageous when dealing with intricate data interactions. Lastly, autoencoders serve as robust denoising tools, enhancing the quality of our analysis, especially with noisy datasets. 

Autoencoders consist of two primary components: an encoder and a decoder, both of which are trained to reconstruct input data. The encoder transforms the input data into a lower-dimensional latent space representation, while the decoder maps this latent representation back to the original input space. The primary objective of an autoencoder is to minimize the reconstruction error between the input data and the reconstructed output, all the while reducing the dimensionality of the latent space representation. In the course of development, autoencoders have been adapted for use with intentionally modified images, such as those containing noise removal. In this context, autoencoders are employed to minimize the error between the reconstructed output and a reference image, a process that can be expressed as follows:

(1) $$L\left(x,\hat{x}\right)=\|x-{\hat{x}\|}^{2}+\alpha {\left\|z\right\|}^{2}$$

where *x* and $\hat{x}$, respectively, are the label and reconstructed images. $\|x-{\hat{x}\|}^{2}$ is the squared L2 norm, α is the hyperparameter that controls the weight of the regularization term, and ‖z‖ is the latent space representation.

**Figure 4:**
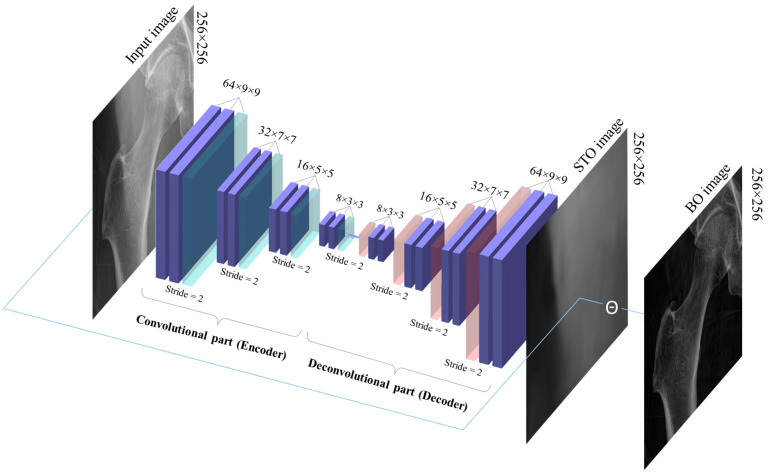
Architecture of the convolutional autoencoder model for enhancing the X-ray of the hip.

In this study, the autoencoder is a convolutional feedforward neural network, also known as a convolutional autoencoder. The input and output layer of the network represents the input data and the reconstructed output, respectively. The encoder consists of many convolutional layers, and the decoder is a mirror image of the encoder, having convolutional layers that increase in size until reaching the output layer. The latent space representation is typically the output of any intermediate layer of the encoder, as shown in Figure 4.

The encoder and decoder are concurrently trained through the iterative process of backpropagation, being aimed at minimizing the reconstruction error between the input data and the reconstructed output. This reconstruction error is typically quantified using the mean squared error (MSE) computed between the input data and the reconstructed output. The dimensionality of the latent space representation is regulated based on the number of neurons within the intermediate layer of the encoder.

After reconstructing the STO image from the input X-ray of the hip by applying the trained autoencoder, the original X-ray of the hip was subtracted from the output image, which finally provided the image representing the X-ray absorption of bone tissue.

### 3.3. Keypoint Detection for Region Partitioning

To extract the feature from the X-rays of the hip, partitioning the area of important hip sections, particularly the three regions including neck, troch, and inter-troch regions, is necessary. To perform this task, the decentralized CNN [18,19] is applied for accurately locating multiple landmarks, which are used to define the boundaries between hip regions. This method narrows the ROI according to each order, which not only reduces the number of unrelated features that can affect the results but also improves the diversity of the training dataset.

**Figure 5:**
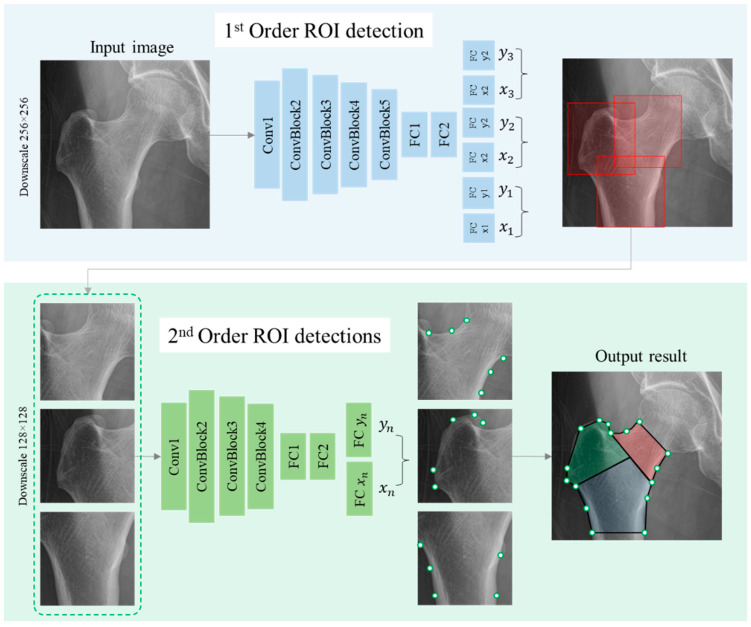
Decentralized landmark detection CNN models for partitioning areas of hips.

Firstly, the CNN model in the first order detects the center points of three ROIs representing three regions of hips. The input data size was standardized into 256 × 256 pixels. The architecture of the trained first-order CNN is as shown in Figure 5, with six outputs representing the horizontal and vertical coordinate values of center points. Continuously, three ROIs are cropped and resized into 128 × 128 pixels, which are considered to be the input for the second-order CNN. With the number of detected landmarks depending on the region of the hip considered, particularly six landmarks for the neck and five for the troch and inter-troch regions, the second-order CNN models accurately locate the required key points, as shown in Figure 5. Consequently, the landmarks detected with respect to each region are used to define the boundary of the area for extracting features.

The weight factors of the trained CNN models are adjusted based on the difference between the output $A^{i}$ obtained from the deep-learning model containing the calculated position value, i for the input image, and the label *Y^i^* created based on the actual position of the required points in the input image. The difference in *L* is calculated using the mean square error loss function stated in Equation (2), which is useful for handling features corrupted by outliers, where *n* denotes the number of labels in the dataset.

The weight factors of the trained CNN models are adapted according to the disparity between the output, denoted as $A^{i}$, generated via the deep-learning model, incorporating the calculated position value i, for the input image, and the corresponding label $Y^{i}$, derived from the actual positions of the required points within the input image. The discrepancy L is computed utilizing the mean squared error loss function, as expressed in Equation (2), which proves effective at addressing features affected by outliers. Here, *n* signifies the count of labels in the dataset.

(2) $$L=\frac{\sum_{i=1}^{n}{\left(A^{i}-Y^{i}\right)}^{2}}{n}$$

After every training iteration, the weight factor, denoted as $\theta_{t}$, undergoes an update for the ${\left(t+1\right)}^{th}$ iteration, following the protocol articulated in Equation (3). In this equation, ‘m’ represents the batch size (m = 64), η signifies the learning rate (initially configured at 0.001 and subsequently adjusted utilizing SGDM with a momentum of 0.95) [20], and ∇ denotes the gradient operator.

(3) $$\theta_{t+1}=\theta_{t}-\eta \nabla_{\theta_{t}}[L(A_{m},Y_{m})]$$

### 3.4. Thresholds for BMD Prediction

From the partitioned areas of the hip, multiple features are calculated and loaded into the linear regression model for predicting BMD values. The calculated values are the mean grayscale value of all voxels inside of each partitioned area. In terms of the pairs of the thresholds $\left[T_{1},T_{2}\right]$, the values are selected based on the distribution of the grayscale values G in the partitioned hip areas. In particular, there are three pairs of thresholds, which, respectively, are as follows:

Threshold 1: [mean(G)−std(G),mean(G)+1.96×std(G)],

Threshold 2: [mean(G)−1.96×std(G),mean(G)+1.96×std(G)],

Threshold 3: [mean(G)−1.96×std(G),mean(G)+std(G)]

**Figure 6:**
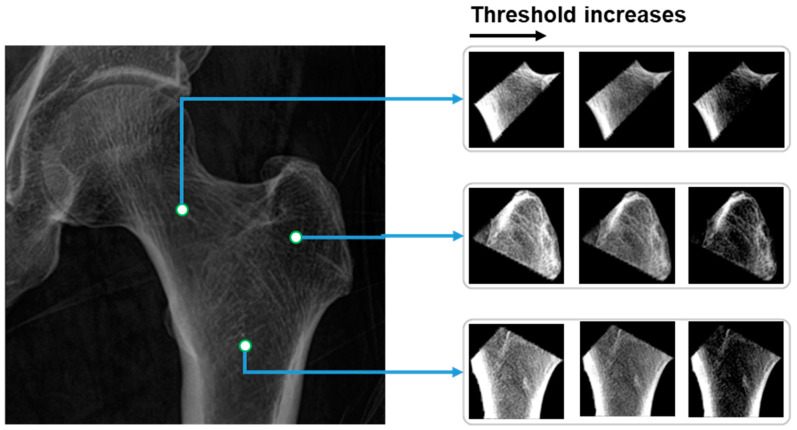
Three hip regions under three thresholds of grayscale values.

Consequently, before normalizing and calculating the mean grayscale value, the grayscale values in hip regions G are first narrowed based on multiple pairs of thresholds $\left[T_{1},T_{2}\right]$, as shown in Equation (4):

(4) $$\begin{array}{c} If\;G\left(i,j\right)<T_{1},\;\;\;set\;G\left(i,j\right)=T_{1}\;\\ If\;G\left(i,j\right)>T_{2},\;\;\;set\;G\left(i,j\right)=T_{2} \end{array}$$

where (i, j) are the coordinates of the pixel in the image, and G is the grayscale value of the hip areas. The image of hip regions after applying the thresholds and normalizing is as shown in Figure 6.

Moreover, in alignment with the findings of Newton-John and Morgan [15] regarding the correlation between biological parameters, namely age, height, and weight, and BMD, these data are subjected to normalization and subsequently used as inputs for the linear regression model, facilitating the prediction of BMD.

## 4. Results

### 4.1. Efficiency of Image Enhancement with Convolutional Autoencoder

After obtaining the features, including the calculated features from images and the biological parameters, they are loaded into a linear regression model for predicting the BMD values. The BMD values measured from DXA are used as the ground truth values for comparison. 

**Figure 7:**
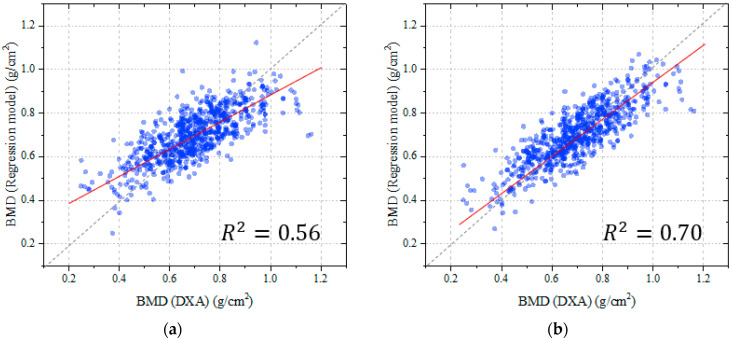
The correlation between the BMD values from DXA and the proposed method: (**a**) without applying the enhancement method; (**b**) with applying the enhancement method.

To validate the efficiency of the proposed image enhancement method on the accuracy of the predicted BMD, comparing the utilization of original X-ray image, the correlation between the predicted and ground truth BMDs of two scenarios with and without applying the enhancement method are determined and shown in Figure 7. Visually, it can be recognized that, with the correlation coefficient of 0.83 (p<0.001) in the case of using the enhancement method, there is a significant improvement in terms of the accuracy compared to the value of 0.72 (p<0.001) recorded using the original image as the input.

In addition, it can be seen in Figure 7a that in case of using the original image, compared to the BMD from DXA, there are many points that represent high deviation between the predicted BMD and the ground truth. These are mainly attributed to obesity patients with large amounts of fat tissue affecting the clarity of the X-rays and, consequently, the BMD measurement. This effect was remarkably refined with the proposed approach using the autoencoder technique to extract the soft tissue absorption, with the decrease in outlier points being the result in Figure 7b.

### 4.2. K-Fold Cross Validation for BMD Prediction Results

To estimate the accuracy of the trained linear regression model, k-fold cross-validation, which is a powerful technique that can help to ensure that the machine learning model is not overfitting to the training data and provides a more reliable estimate of the model’s performance, is utilized. With selected k=10, the model is then trained on k−1=9 of the folds and tested on the remaining fold. This process is repeated k=10 times, each time using a different fold as the test set and the remaining folds as the training set. The results are then all collected to give an estimate of the model’s performance. In this validation, the performance of the proposed method in the case of using the original image and enhanced image as the inputs is separately validated.

**Figure 8:**
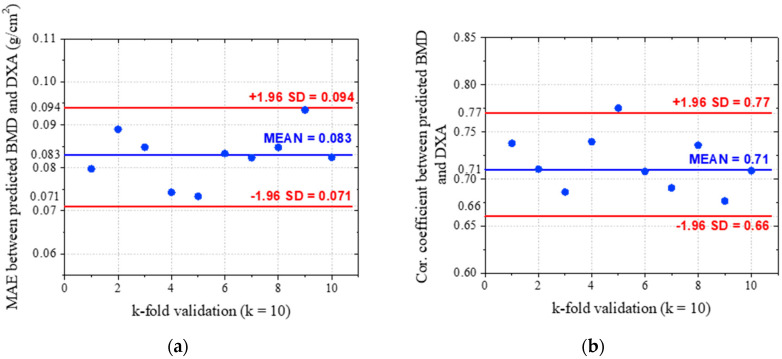

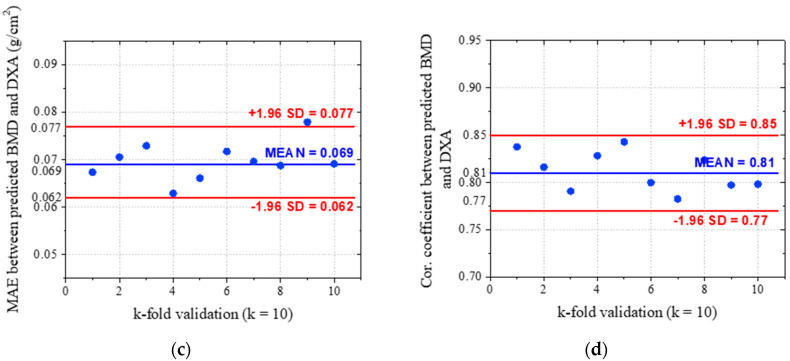
Bland–Altman plot of k-fold cross validation for BMD prediction compared to DXA method: (**a**) correlation coefficient with original image; (**b**) MAE with original image; (**c**) correlation coefficient with enhanced image; (**d**) MAE with enhanced image.

Figure 8 shows the validation result of the proposed method after 10-fold with the metrics of correlation coefficient and the mean absolute error (MAE). In particular, Figure 8a,b sequentially demonstrates the MAE of 0.083 g/cm^2^ and correlation coefficient of 0.71 in the case of using the original X-ray of the hip as the input. With a correlation coefficient of 0.81 and MAE of 0.069 g/cm^2^, respectively, as shown in Figure 8c,d, the efficiency is consistently highlighted.

In addition, the deviation between validation folds of tests with and without using enhancement methods has a significant difference. In detail, the 1.96× standard deviation of the MAE using original image is 0.011, which is larger than the value of 0.008 recorded using the enhanced image. These results can also be observed in the metrics of the correlation coefficient, with the values of 1.96× standard deviation with and without enhancement method, respectively, being 0.06 and 0.04.

To validate the performance of the trained linear regression model for predicting BMD, the averaged correlated coefficient of 10 folds is 0.81 (p<0.001), which shows a similar tendency compared to the fitting result of 0.83 (p<0.001), consequently demonstrating the non-existence of overfitting. 

### 4.3. Efficiency of Using Multiple Thresholds for Extracting Features

**Figure 9:**
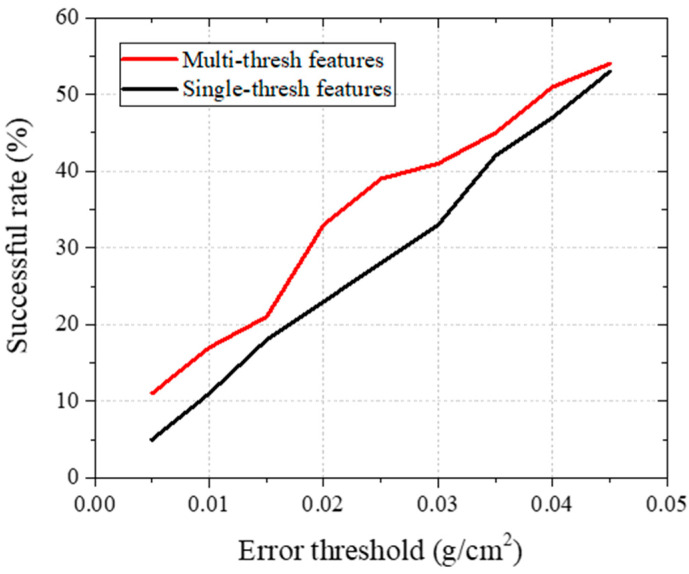
Comparison of success rates when using one or multiple thresholds for extracting features.

In the step for feature extraction, before calculating the mean grayscale value of the hip regions, three threshold sets are applied to standardize the range of the grayscale values. The utilization of three threshold sets is aimed at providing information about X-ray absorption in more detail compared to using a single threshold set. To demonstrate the efficiency of this proposed solution, the success rate with respect to the error levels is recorded for both single threshold sets of [mean(G)−1.96×std(G), mean(G)+1.96×std(G)] and the developed solution with features from multiple thresholds. It can be recognized in Figure 9 that the proposed approach achieved a higher success rate for an error threshold from 0 to 0.045 g/cm^2^. In other words, using multiple thresholds for extracting features in predicting BMD helps to reduce the errors compared to using the DXA method, which proves its efficiency in terms of increasing the accuracy of the proposed method.

## 5. Discussion

The measurement of bone mineral density (BMD) holds paramount importance in the diagnosis of osteoporosis, assessment of fracture risk prediction, monitoring of osteoporosis treatment efficacy, and evaluation of overall bone health. In the scope of this research, we have introduced an automated method for BMD measurement from X-ray images. A novel aspect of our research lies in the utilization of convolutional autoencoders to extract grayscale values corresponding to soft tissue absorption. This process aids in the segmentation of bone tissue absorption from the original image, subsequently yielding the input image for BMD measurement. It is based on the landmark system, which is automatically detected by applying decentralized CNN. The areas of the three hip regions partitioned were then subjected to multiple thresholds to extract features. Finally, the grayscale features combined with the biological parameters are considered as the inputs for the linear regression model to predict the BMD value.

Deep learning has emerged as a promising trend in the field of artificial intelligence for the diagnosis of osteoporosis using X-ray images. This advancement holds the potential to empower healthcare professionals to assess bone health without the need for costly specialized equipment. Previous studies in this field have mainly focused on the classification of the presence of osteoporosis or fractures in the input hip X-ray images [21,22,23,24]. The diagnostic techniques used to identify fractures are often considered bothersome as the symptoms are readily noticeable, causing significant discomfort and disability. In addition, medical professionals find it challenging to determine the severity of osteoporosis and determine the most appropriate treatment and preventative measures using automated classification methods. Therefore, the approach outlined in this study concentrates on evaluating BMD from X-ray images, which is currently the standard indicator for assessing osteoporosis.

Fathima et al. [25] introduced a modified U-Net model designed specifically for the efficient segmentation of bone diseases within DXA and X-ray images. It is worth noting that while U-Net is widely recognized for its efficacy in medical image segmentation tasks, its suitability for diagnosing specific diseases such as osteoporosis may be somewhat limited. In a related context, Zheng et al. [26] proposed an algorithm aimed at training a regression model for bone mineral density (BMD) utilizing convolutional neural networks (CNN) applied to X-ray images. Their methodology incorporated labeled images containing DEXA-measured BMD values and unlabeled images augmented with pseudo-BMDs. To enhance the precision of BMD regression, they introduced an innovative adaptive triplet loss technique. Furthermore, Geng et al. [27] conducted an analysis of X-ray images to identify pathological bone conditions, leveraging deep convolutional neural networks (DCNN) to expedite diagnostic assessments. However, it is important to consider that the utilization of regression-based CNN models for predicting BMD in X-ray images, particularly when accounting for the influence of soft tissues, as explored in this research, may be deemed somewhat simplistic and might not yield a significant improvement in prediction performance.

Yasaka et al. [28] employed a convolutional neural network (CNN) to estimate bone mineral density (BMD) from CT images, achieving a notable correlation coefficient of 0.84 (p<0.001) when compared to DXA results. These compelling outcomes can be attributed to the choice of input CT images, which enable the visualization of inner bone structure and density, thereby explaining the superior efficiency of CT images compared to X-rays. In a related study, Sato et al. [14] introduced a deep learning-based method for BMD measurement from X-rays. Their results were also compared to DXA measurements, yielding a correlation coefficient of approximately 0.75 for hip BMD prediction, a figure closely resembling the outcome of our research, even without the use of the image enhancement techniques employed in our study. Nguyen et al. [13] also introduced a method that was the combination of a CNN and the Sobel algorithm for extracting the trabecular pattern on the hip X-ray for measuring BMD. While the obtained result yielded a correlation coefficient of 0.808 (p<0.0001), it is important to acknowledge that the dataset used in this study intentionally excluded X-ray images with a pronounced impact of soft tissue, which was a deliberate choice made for quality control purposes. Nonetheless, this deliberate exclusion has implications for the practical applicability of our method, as standard hip X-ray images typically encompass the grayscale values associated with soft tissue absorption. Hsieh et al. [29] presented an interesting method to automatically locate the position of the hip based on the detected landmarks, then measuring the BMD using the linear regression. With the advantages of large number of datasets collected, the BMD measured via the developed method reached a correlation coefficient of 0.93 compared to the DXA technique. However, there is no mention of the effects of the X-ray image quality on the prediction results. High accuracy predictions can be obtained using quality-controlled X-ray sets that exclude cases with an uneven distribution of soft tissue. However, the practical applicability of the method may not match the demonstrated results due to the inevitable uneven distribution of soft tissue in various states. Therefore, as a validation result, our proposed method offers a solution to improve the accuracy of BMD predictions on X-rays by filtering out soft tissue and focusing only on the bone tissue.

The usage of an autoencoder, which takes X-rays as inputs and regenerates the image, increases the total processing time for predicting BMD up to 1.4 s. Although this is slightly higher than the time taken in other studies, which mainly used X-ray images for detecting landmarks and then predicting BMD, the advantage of measuring BMD on X-rays in terms of productivity is still undeniable compared to the DXA method. DXA typically takes around 10 to 20 min to complete a single case. 

Typically, with regard to X-ray devices, the generator affords radiology technologists control over three crucial technical factors: the tube voltage applied across the X-ray tube, the tube current passing through the X-ray tube, and the total exposure duration during which the current flows. These parameters significantly influence the characteristics of the obtained X-ray images, impacting factors such as contrast and noise. In our research, a consistent device configuration was employed for image acquisition across all instances. Nevertheless, the potential impact of varying device configurations on prediction accuracy remains unexplored, thus representing a noteworthy limitation of this study. Further investigation into this aspect is warranted to gain a comprehensive understanding of its implications. This research also faces limitations in establishing connections with clinical institutions and obtaining the necessary pQCT datasets, which is a time-consuming process. Additionally, the validation dataset lacks key bone markers relevant to osteoporosis. In response to this issue, future work will focus on collaborating with suitable institutions, collecting the requisite data, and incorporating bone markers into the validation process. These efforts are vital to ensure the effectiveness and reliability of our proposed method in addressing the complexities of osteoporosis detection and assessment.

## 6. Conclusions

In this study, we have introduced a fully automated approach for predicting hip bone mineral density (BMD), utilizing conventional X-ray images as input. The principal innovation of this method, distinguishing it from prior research that employed original X-rays as inputs, lies in the utilization of the proposed convolutional autoencoder technique to selectively isolate soft tissue absorption from X-ray images. To generate training data for the convolutional autoencoder model, we devised a method to synthesize images from regions exhibiting soft tissue and bone tissue absorption at various positions within conventional hip X-rays. The aim was to obtain a new image featuring only bone tissue absorption, with the intent of enhancing BMD prediction accuracy following the mitigation of soft tissue effects. The effectiveness of our proposed method was validated through a comparative analysis via the DXA method. Notably, the autoencoder-enhanced image exhibited superior performance compared to those of the results obtained from the original X-ray images. With a processing speed of 1.4 s, and considering the widespread use of X-rays, our method presents a cost-effective alternative for patients and institutions unable to invest in expensive DXA equipment. It has the potential to assume a central role in bone health monitoring and osteoporosis diagnosis in the future. 

## Figures and Tables

**Table 1:** Statistical data for participating patients.

Parameters	Patient Gender	
	Female	Male
Number of cases	801	138
Average age (years) ± STD	68.5 ± 15.11	69.18 ± 13.68
Mean body height ± STD (cm)	153.27 ± 8.84	166.29 ± 6.47
Mean body weight ± STD (kg)	57.63 ± 11.67	65.52 ± 9.40
Mean bone mineral density ± STD (g/cm^2^)	0.7 ± 0.15	0.79 ± 0.15

## Data Availability

The data used in this study is not available for public sharing due to privacy and confidentiality considerations.

## References

[1] Lyritis G.P. (1996). The MEDOS Study Group. Epidemiology of hip fracture: The MEDOS study. Osteoporos. Int..

[2] Myers A.H., Robinson E.G., Van Natta M.L., Michelson J.D., Collins K., Baker S.P. (1991). Hip fractures among the elderly: Factors associated with in-hospital mortality. Am. J. Epidemiol..

[3] Poór G., Atkinson E.J., Lewallen D.G., O’Fallon W.M., Melton L.J. (1995). Age-related hip fractures in men: Clinical spectrum and short-term outcomes. Osteoporos. Int..

[4] Parker M., Johansen A. (2006). Hip fracture. BMJ.

[5] Singh A., Dutta M.K., Jennane R., Lespessailles E. (2017). Classification of the trabecular bone structure of osteoporotic patients using machine vision. Comput. Biol. Med..

[6] Koot V.C.M., Kesselaer S.M.M.J., Clevers G.J. (1996). Evaluation of the Singh Index for Measuring Osteoporosis. J. Bone Jt. Surg. Br..

[7] Hauschild O., Ghanem N., Oberst M., Baumann T., Kreuz P.C., Langer M., Suedkamp N.P., Niemeyer P. (2009). Evaluation of Singh index for assessment of osteoporosis using digital radiography. Eur. J. Radiol..

[8] Hübsch P., Kocanda H., Youssefzadeh S., Schneider B., Kainberger F., Seidl G., Kurtaran A., Gruber S. (1992). Comparison of dual energy x-ray absorptiometry of the proximal femur with morphologic data. Acta Radiol..

[9] Klatte T.O., Vettorazzi E., Beckmann J., Pueschel K., Amling M., Gebauer M. (2015). The Singh Index does not correlate with bone mineral density (BMD) measured with dual energy X-ray absorptiometry (DXA) or peripheral quantitative computed tomography (pQCT). Arch. Orthop. Trauma Surg..

[10] El Maghraoui A., Roux C. (2008). DXA scanning in clinical practice. QJM.

[11] Pulkkinen P., Jamsa T., Lockmuller E.M., Partanen J., Koski J.M. (2008). Experimental hip fracture load can be predicted from plain radiography by combined analysis of trabecular bone structure and bone geometry. Osteoporos. Int..

[12] Liu Y.C., Lin Y.C., Tsai P.Y., Lin C.C., Hsu H.C., Tseng V.S. (2020). Convolutional neural network-based humerus segmentation and application to bone mineral density estimation from chest X-ray images of critical infants. Diagnostics.

[13] Nguyen T.P., Chae D.S., Park S.J., Yoon J. (2021). A novel approach for evaluating bone mineral density of hips based on Sobel gradient-based map of radiographs utilizing convolutional neural network. Comput. Biol. Med..

[14] Sato Y., Yamamoto N., Inagaki N., Iesaki Y., Asamoto T., Suzuki T., Takahara S. (2022). Deep Learning for Bone Mineral Density and T-Score Prediction from Chest X-rays: A Multicenter Study. Biomedicines.

[15] Newton-John H.F., Morgan D.B. (1970). The loss of bone with age, osteoporosis, and fractures. Clin. Orthop. Relat. Res..

[16] Robin J.C., Kelly R.S., Mannley N., Thomas C.C., Ambrus J.L. (1978). Studies on osteoporosis I. Experimental models. Effect of age, sex, genetic background, diet, steroid and heparin treatment on calcium metabolism of mice. Res. Commun. Chem. Pathol. Pharmacol..

[17] Keaveny T.M., Yeh O.C. (2002). Architecture and trabecular bone—Toward an improved understanding of the biomechanical effects of age, sex and osteoporosis. J. Musculoskelet. Neuronal Interact..

[18] Nguyen T.P., Chae D.S., Park S.J., Kang K.Y., Lee W.S., Yoon J.H. (2020). Intelligent analysis of coronal alignment in lower limbs based on radiographic image with convolutional neural network. Comput. Biol. Med..

[19] Chae D.S., Nguyen T.P., Park S.J., Kang K.Y., Won C.H., Yoon J.H. (2020). Decentralized convolutional neural network for evaluating spinal deformity with spinopelvic parameters. Comput. Methods Prog. Biomed..

[20] Jayalashmy S., Sudha G.F. (2020). Scalogram based prediction model for respiratory disorders using optimized convolutional neural networks. Artif. Intell. Med..

[21] Singh M., Riggs B.L., Beabout J.W., Jowsey J. (1972). Femoral trabecular-pattern index for evaluation of spinal osteoporosis. Ann. Intern. Med..

[22] Lee J.H., Hwang Y.N., Park S.Y., Jeong J.H., Kim S.M. (2015). Diagnosis of osteoporosis by quantification of trabecular microarchitectures from hip radiographs using artificial neural networks. J. Comput. Theor. Nanosci..

[23] Yu X., Ye C., Xiang L. (2016). Application of artificial neural network in the diagnostic system of osteoporosis. Neurocomputing.

[24] Jang R., Choi J.H., Kim N., Chang J.S., Yoon P.W., Kim C.-H. (2021). Prediction of osteoporosis from simple hip radiography using deep learning algorithm. Sci. Rep..

[25] Fathima S.N., Tamilselvi R., Beham M.P., Sabarinathan D. (2020). Diagnosis of Osteoporosis using modified U-net architecture with attention unit in DEXA and X-ray images. J. X-Ray Sci. Technol..

[26] Zheng K., Wang Y., Zhou X.-Y., Wang F., Lu L., Lin C., Huang L., Xie G., Xiao J., Kuo C.-F. (2021). Semi-supervised learning for bone mineral density estimation in hip X-ray images. arXiv.

[27] Geng Y., Liu T., Ding Y., Liu W., Ye J., Hu L., Ruan L. (2021). Deep learning-based self-efficacy X-ray ımages in the evaluation of rheumatoid arthritis combined with osteoporosis nursing. Sci. Prog..

[28] Yasaka K., Akai H., Kunimatsu A., Kiryu S., Abe O. (2020). Prediction of bone mineral density from computed tomography: Application of deep learning with a convolutional neural network. Eur. Radiol..

[29] Hsieh C.I., Zheng K., Lin C., Mei L., Lu L., Li W., Chen F.P., Wang Y., Zhou X., Wang F. (2021). Automated bone mineral density prediction and fracture risk assessment using plain radiographs via deep learning. Nat. Commun..

